# Non-invasive assessment of the reproductive cycle in free-ranging female African elephants (*Loxodonta africana*) treated with a gonadotropin-releasing hormone (GnRH) vaccine for inducing anoestrus

**DOI:** 10.1186/1477-7827-10-63

**Published:** 2012-08-25

**Authors:** Gabriela Benavides Valades, Andre Ganswindt, Henry Annandale, Martin L Schulman, Henk J Bertschinger

**Affiliations:** 1 Department of Production Animal Studies, Faculty of Veterinary Science, University of Pretoria, Onderstepoort, RSA; 2 Mammal Research Institute, Department of Zoology and Entomology, University of Pretoria, Pretoria, RSA; 3 Onderstepoort Veterinary Academic Hospital, Faculty of Veterinary Science, University of Pretoria, Onderstepoort, RSA

**Keywords:** GnRH immunocontraception, African elephant cows, Oestrous cycle, Non-invasive monitoring, Faecal progestagen metabolites, Ecological effects

## Abstract

**Background:**

In southern Africa, various options to manage elephant populations are being considered. Immunocontraception is considered to be the most ethically acceptable and logistically feasible method for control of smaller and confined populations. In this regard, the use of gonadotropin-releasing hormone (GnRH) vaccine has not been investigated in female elephants, although it has been reported to be safe and effective in several domestic and wildlife species. The aims of this study were to monitor the oestrous cycles of free-ranging African elephant cows using faecal progestagen metabolites and to evaluate the efficacy of a GnRH vaccine to induce anoestrus in treated cows.

**Methods:**

Between May 2009 - June 2010, luteal activity of 12 elephant cows was monitored non-invasively using an enzyme immunoassay detecting faecal 5alpha-reduced pregnanes (faecal progestagen metabolites, FPM) on a private game reserve in South Africa. No bulls of breeding age were present on the reserve prior to and for the duration of the study. After a 3-month control period, 8 randomly-selected females were treated twice with 600 micrograms of GnRH vaccine (Improvac®, Pfizer Animal Health, Sandton, South Africa) 5-7 weeks apart. Four of these females had been treated previously with the porcine zona pellucida (pZP) vaccine for four years (2004-2007).

**Results:**

All 12 monitored females (8 treated and 4 controls) showed signs of luteal activity as evidenced by FPM concentrations exceeding individual baseline values more than once. A total of 16 oestrous cycles could be identified in 8 cows with four of these within the 13 to 17 weeks range previously reported for captive African elephants. According to the FPM concentrations the GnRH vaccine was unable to induce anoestrus in the treated cows. Overall FPM levels in samples collected during the wet season (mean 4.03 micrograms/gram dry faeces) were significantly higher (P<0.002) than the dry season (mean 2.59 micrograms/gram dry faeces).

**Conclusions:**

The GnRH vaccination protocol failed to induce anoestrus in the treated female elephants. These results indicate that irregular oestrous cycles occur amongst free-ranging elephants and are not restricted to elephants in captivity. The relationship between ecological conditions and endocrine activity were confirmed. Free-ranging female elephants were observed to not cycle continuously throughout the year in the absence of adult bulls.

## Background

In southern Africa the savannah elephants (*Loxodonta africana)* began recovering in numbers after the banning of the ivory trade in 1990. Currently 70% of Africa’s elephants occur in southern Africa with an annual rate of increase of 4-5% [1-3]. Many of the smaller elephant populations (<1000 elephants) are held in fenced reserves. The space constraint imposed by the fencing results in: limiting natural behaviour [4], habitat degradation with a consequent negative impact on the many species that share the same space, conflict with human communities that border conservation areas and the possibility of population crashes [5,6].

Elephants are considered to be non-seasonal, polyoestrus and uniparous breeders [7]; although there is a peak in conception rates and births during the rainy season, when resources are more abundant [8-11]. In captive elephants the duration of the oestrous cycle length is 13 to 17 weeks [12]. It consists of a luteal phase (LP) lasting 6 to 12 weeks with elevated levels of progestagens and an inter-luteal or follicular phase (ILP) ranging from 4 to 6 weeks [7,13]. Oestrus with typical behavioural signs lasts 2 to 8 days [14,15].

Several options are being practiced or considered to mitigate the overpopulation problem. These include culling, range expansion through establishment of cross-border protected areas and protection of migration corridors, translocation to under-populated areas and fertility control [16,17]. Among the available fertility control options, immunocontraception using native porcine zona pellucida (pZP) proteins seems to be the most viable means of population management for smaller and confined populations of elephants [18]. A possible disadvantage of pZP-immunocontraception is that treated females continue to cycle normally, approximately every 15 weeks [19] and continuously attract bulls which could potentially disrupt the herd [20].

An additional potentially-promising option for fertility control of free-ranging animals is immunization against GnRH. This has been safely and effectively used in several domestic and wildlife species including white-tailed deer (*Odocoileus virginianus*), feral and domestic swine (*Sus scrofa*), cattle (*Bos primigenius*), squirrels (*Spermophilus beecheyi)*, rats (*Rattus norvegicus)*, feral and domestic horses (*Equus caballus)*[21-25]. The vaccine stimulates the production of anti-GnRH antibodies that bind to endogenous GnRH and prevent the molecules from binding to receptors on the pituitary gonadotrophs [26,27]. As a consequence the down-stream release of the gonadotrophic hormones LH and FSH is reduced which results in down-regulation of spermatogenesis in the male and failure of follicle development and ovulation in the female. This leads to gonadal regression and suppression of sexual behaviour in treated animals returning them to a prepubertal state [24,28-30]. This contraceptive effect lasts from 1 to 3 years in horses [24,26], 6 months in cattle [31], 2 years in white-tailed deer [21], and 1 to 2 years in bison (*Bison bison)*[32]. In African elephant bulls the GnRH vaccine has proven to be a safe method to suppress musth and androgen related aggressive behaviour [33]. In female elephants GnRH vaccine might offer an alternative approach to the present immunocontraceptive method which uses native pZP proteins. From a biological perspective, the GnRH vaccine offers the advantage of inducing anoestrus. This is more reflective of the situation in the wild where cows spend 50% of post-pubertal life in pregnancy anoestrus and most of the remaining time in what is presumed to be lactation anoestrus [33-35]. This is based on an inter-calving interval of 4-5 years and a gestation period of 22 months [20]. Other advantages of GnRH vaccines include commercial availability, ease of storage and reversibility once antibody titers decline [21,22,25].

The overall aim of this study was to monitor luteal profiles of free-ranging African elephant (*Loxodonta africana*) females treated with GnRH vaccine. More specifically, (i) to monitor the reproductive status and oestrous cycle of 12 females (8 treated and 4 control) through non-invasive faecal progestagen metabolite (FPM) evaluation using an enzyme immunoassay detecting faecal 5*α*-reduced pregnanes, (ii) to assess the efficacy of the GnRH vaccine to induce anoestrus, (iii) to assess the seasonal influence of ecological conditions on FPM concentrations, and (iv) to record signs of oestrus pre- and post-GnRH vaccine administration.

## Methods

### Study site

The Entabeni Private Game Reserve (EPGR) is situated in the Waterberg ecological region of the Limpopo Province, South Africa (28° 39’ S, 24° 11’ E). The reserve is divided into two separate sections: Upper Escarpment (3900 ha and 1500 m above sea level) and Lower Escarpment (7300 ha and 1150 m above sea level). EPGR is a summer rainfall area with an average annual rainfall of 589 mm mainly falling from October to March [36]. In this study, rainfall was monitored daily from May 2009 to June 2010, substantial rainfall (>50 mm per month) was recorded between October 2009 to January 2010, as well as March and April 2010. The vegetation of the area is described as sour bushveld [37], with nutritionally poorer vegetation on the Upper Escarpment compared to the Lower Escarpment [Entabeni Private Game Reserve Veld Condition Assessment, February-April 2008; Spaan, R unpublished data].

### Elephant populations

Between 2008-2010, the total EPGR elephant population consisted of 13 females and three male calves free-roaming within two fenced areas. There were 9 females and one calf on the Lower Escarpment whereas the Upper Escarpment population consisted of 4 females and 2 calves (Table 1). No sexually mature bulls [20] were present in either section of the EPGR during the two years preceding and for the duration of the study. The 12 females of reproductive age (≥10 years old) [20] were included in the study (Table 1). The Lower Escarpment herd was translocated to EPGR in July 2008 from the Shambala Game Reserve, South Africa, where the four oldest females (Table 1) were treated with pZP vaccine from 2004 to 2007 [20]. The Upper Escarpment herd was translocated from Kruger National Park in 1998 together with two adult bulls that were later culled in 2007. None of the females had received any contraceptive therapy at any time. Identification kits, comprising sketches of the ear patterns with corresponding photographs and other obvious characteristics, were made of all EPGR elephants thus allowing for easy individual identification during the study [16,38]. Age was determined from known dates of birth of younger animals and rough age estimates based on shoulder heights and sizes of older animals were made [39] (Table 1). This study was conducted under approval and in accordance with the guidelines of the University of Pretoria Animal Use and Care Committee (V015-09).

**Table 1:** Mean faecal progesterone metabolite concentrations ± SD of female African elephants

**ID**	**Age (years)**	**Herd**^**a**^	**Treatment**^**b**^	**Number of faecal samples collected**	**Mean FPM**^**c**^**concentrations (μg/g DW)**		
					**Baseline**^**d**^	**Luteal phase**^**d**^	**Inter-luteal phase**^**d**^
*1*^*e*^	30+	LE	T	64	1.96 ± 0.86	4.83 ± 3.44	1.27 ± 0.34
*9*^*e*^	30+	LE	T	56	3.03 ± 1.16	6.32 ± 3.55	2.09 ± 0.64
*2*^*e*^	25	LE	T	64	3.13 ± 1.39	6.18 ± 3.35	2.03 ± 0.76
*8*^*e*^	20	LE	T	50	2.63 ± 1.23	5.47 ± 2.76	1.64 ± 0.42
*3*	18	LE	T	59	3.10 ± 1.30	6.13 ± 3.50	1.93 ± 0.69
*5*	15	LE	T	59	1.81 ± 0.75	4.28 ± 2.04	1.30 ± 0.35
*4*	12	LE	C	59	2.34 ± 1.30	5.84 ± 3.57	1.33 ± 0.54
*7*	10	LE	C	63	1.53 ± 0.55	3.80 ± 2.73	1.10 ± 0.27
*11*	30+	UE	T	25	2.90 ± 1.25	11.12 ± 15.94	1.96 ± 0.58
*12*	30+	UE	T	27	2.39 ± 1.13	11.33 ± 17.75	1.70 ± 0.43
*13*	15	UE	C	20	3.36 ± 1.25	5.89 ± 2.56	2.54 ± 0.75
*14*	15	UE	C	20	2.82 ± 1.25	8.28 ± 6.37	1.83 ± 0.59

^a^*LE* = Lower Escarpment; *UE* = Upper Escarpment.

^b^*T* = GnRH treatment; *C* = Control.

^c^*FPM* = faecal progestagen metabolite.

^d^mean ± SD.

^e^pZP treatment 2004-2007.

### Vaccination of elephants with GnRH

Eight adult females (six from the Lower Escarpment herd and two from the Upper Escarpment herd) received two treatments of the commercially available GnRH-vaccine Improvac® (Pfizer Animal Health, Sandton, South Africa). The target animals received a primary vaccination containing 600 μg GnRF-protein conjugate (3 ml) on September 2009, followed by a single booster with the same dose 5 to 7 weeks later.

The primary vaccination was administered by remote delivery from the ground with a Dan-Inject® darting system (Dan-Inject® International, Denmark) using 60 mm needles thus ensuring deep intramuscular injection. The booster vaccination was administered either from the ground using the same darting system or from a helicopter using a Pneu-Dart® rifle and disposable darts (50 mm needles).

### Observations

All adult individuals of the EPGR elephant population were tracked daily during a 14-month period (May 2009 to June 2010) starting with a three-month control period prior to vaccination. The Lower Escarpment elephants were located via VHF radio telemetry and, once located, were lured into a clearing with supplementary feed. The Upper Escarpment herd was found with conventional tracking methods and often located at a particular feeding spot which has been routinely used to provide supplementary feed during the winter months. The treated animals were observed daily from a distance to determine local side-effects of the vaccination, as well as for signs of lameness or illness [40,41]. Observations of the herds were performed using *ad libitum* sampling [42] and included recordings of reproductive behaviour such as signs of behavioural oestrus like sniffing of the genital orifice or urine by another female and the presence of a vaginal discharge [9,14,43].

### Faecal sample collection and extraction

From May 2009-June 2010 a total of 566 faecal samples were collected from the 12 study animals. Samples were collected from each female elephant once or twice a week [19,44], dependant on sample availability, with an average of 4.23 samples per month per individual for the Lower Escarpment herd and 1.64 for the Upper Escarpment herd. Samples were taken as soon as possible after defecation to ensure positive identification of the individual, with an average collection time of 37.2 min and a maximum of 183 min post-defecation. Using rubber gloves, approximately 10 g of well mixed faecal material per sample was collected. The material was stored in a sealed glass vial, frozen immediately and stored at -20°C until extraction [45].

The collected material was freeze-dried, pulverized and sieved through a metallic mesh to remove fibrous material. Approximately 0.05 g of the powder was mixed with 3 ml of 80% aqueous ethanol [46]. Steroids were extracted by shaking for 15 min on a multi-tube vortex following. The mixture was centrifuged at 1500 g [47] and the supernatant stored at -20°C until hormone analysis.

### Enzyme immunoassay for progestagen metabolites

FPM concentrations were determined using an enzyme immunoassay (EIA) for 5α-pregnan-3β-ol-20-on which has been shown to provide reliable information on reproductive steroid hormone patterns in female elephants [19,48]. EIAs were performed following the methodology of Ganswindt *et al.* ([2002) [49]. The cross-reactivity of the antibody used is described by Szdzuy *et al.* ([2006) [48]. The sensitivity of the assay at 90% binding was 3 pg per well. Inter- and intra-assay coefficients of variation ranged between 3.6% and 12.2%, respectively.

### Data analysis

Definition of the oestrous cycle was based on FPM profiles expressed as μg/g dry faecal weight (DW) and plotted against time (weeks). For each of the 12 monitored females, baseline FPM concentrations were calculated using an iterative process as described by Brown *et al.* ([1999) [50]. Subsequently, the length of occurring ovarian cycles were determined according to the procedure described by Brown *et al.* ([2001) [51], by combining the length of a respective luteal phase and subsequent inter-luteal phase. In addition, mean and standard deviation of FPM concentrations were calculated on an individual level for each phase. In this regard, the increase in FPM concentration following an inter-luteal phase indicates the ovulatory period which has been reported to coincide with maximum male interest and mating [43,52]. Signs of behavioural oestrus were compared with the occurrence of increases in FPM levels.

Females were considered to have irregular oestrous cycles when overall cycle length exceeded or fell short of the reported length of 13 to 17 weeks for captive African elephants [7,13]. Individuals were designated to have an irregular oestrous cycle based on extended inter-luteal phase (>9 weeks), shortened luteal phase (<6 weeks), and/or the presence of marked, random fluctuations in FPM concentrations during the respective phases [13]. Periods of anoestrus were defined if an animal had an inter-luteal phase lasting longer than twice the average duration of a normal inter-luteal phase (range 4 to 6 weeks) [7,13].

To test the effect of treatment (GnRH vaccine) on FPM concentrations a Mann–Whitney *U* test was performed to compare luteal phase concentrations (above baseline values) between treated and control females. Baseline means were also compared between treated and control females using a Mann-Withney *U*-test. To test for a possible short-term effect of the vaccine in the females on the Lower Escarpment, FPM luteal phase concentrations of the three months control period (June-September 2009) and FPM levels from three months following vaccination (September-December 2009) were compared using a Mann–Whitney *U* test.

To assess the seasonal influence on FPM concentrations, median hormone levels were calculated per season (“dry season” = May through September 2009 and May through June 2010 and “wet season” = October 2009 through April 2010) of each individual (n = 12) and then analysed using a Wilcoxon Rank Sum Test for paired samples (with continuity correction of 0.5).

The α-level of significance was set at P<0.05 for all tests. Statistical analyses were performed using NCSS Statistical Software ^©^2007 (version 07.1.19) and KyPlot (Version 2.0 beta 13 1997).

## Results

### Administration of the vaccine

The Dan-Inject® and Pneu-Dart® darting systems were both found to be practical systems to deliver the vaccine. The incomplete delivery of two Dan-Inject® booster vaccinations were attributed to the inexperience of the one operator but also possibly due to the viscous nature of the vaccine solution. Behavioural changes observed during and shortly after the darting events included avoidance of the research vehicle, reduced time at feeding spots, increased wariness and animals fleeing easily. These changes, however, quickly faded away and could not be observed a few days post-darting. The post-vaccination effects of the immunization were limited to small local swellings and all resolved after a maximum of two weeks.

### Effect of vaccine treatment on oestrous cycle

All 12 monitored females showed evidence of luteal activity as evidenced by FPM concentrations exceeding the baseline more than once during the study period (Table 1; Additional file 1, Figure S1). None of the treated females showed anoestrus following treatment as was expected. For the Lower Escarpment herd no significant differences in mean luteal phase FPM levels were observed between treatment (median 4.43 μg/g DW, n = 213, p>0.65) and control groups (median 3.66 μg/g DW, n = 75) or baseline FPM concentrations between treatment (median 2.83 μg/g DW, n = 6, p>0.21) and control groups (median 1.93 μg/g DW, n = 2). For the Upper Escarpment herd there was also no difference in mean luteal phase FPM levels between treatment (median 3.97 μg/g DW, n = 37, p>0.29) and control groups (median 4.84 μg/g DW, n = 21) or baseline FPM concentrations between treatment (median 2.64 μg/g DW, n = 2, p>0.35) and control groups (median 3.09 μg/g DW, n = 2).

The test for a short-term effect of the vaccine on the Lower Escarpment females showed a significant difference between the control period (median 1.99 μg/g DW, n = 106, p<0.05) and post-treatment period (3.8 μg/g DW, n = 92) on the luteal concentrations of treated females (n = 6), as well as for the control females (n = 2) (1.42 μg/g DW, n = 35, p<0.05) for the control period and (2.86 μg/g DW, n = 34) for the post-treatment period.

### Oestrous cycle length and component phases

**Figure 1:**
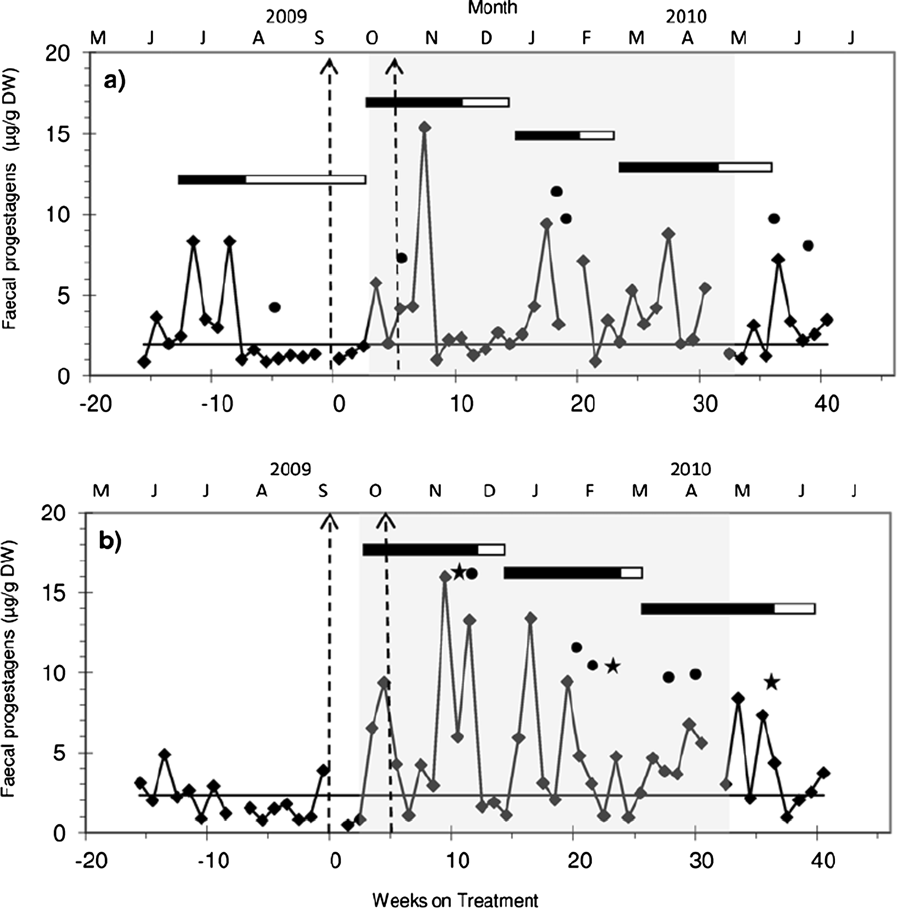
**Faecal progestagen metabolite concentrations for two adult female African elephants.a**) Female No. 1 treated with the GnRH vaccine and **b**) Female No. 4; control group. Solid line represents baseline concentration, horizontal solid bars represent luteal phase, horizontal open bars represent inter-luteal phase, and dotted line arrows correspond to darting dates of primary vaccine and subsequent booster vaccine. Circles stand for sniffing into genital opening or urine and stars for vaginal discharge observations. Wet season is illustrated in grey background.

In total sixteen cycles could be identified in the eight females of the Lower Escarpment herd during the study period (Table 2), but only four of these (the cycles of three treated females) fell within the normal 13 to 17 weeks oestrous cycle range described for African elephants [7,13] (Figure 1a). The mean length of these four cycles was 14.11 ± 1.35 weeks with LP and ILP of 9.39 ± 0.75 and 4.72 ± 1.11 weeks, respectively. The 12 remaining cycles had shorter (n = 3; 3 treatment, 0 control), longer (n = 3; 2 treatment, 1 control) or normal (n = 6; 3 treatment, 3 control) LP combined with shorter (n = 5; 2 treatment, 3 control) or longer than expected (n = 4; 4 treatment, 0 control) ILP (Figure 1b).

**Table 2:** Summary of oestrous cycle lengths in weeks of female African elephants of the Lower Escarpment herd

	**Cycle Phase**^**a**^	**Cow No.**							
		**1**^**b**^	**2**^**b**^	**3**^**b**^	**4**^**c**^	**5**^**b**^	**7**^**c**^	**8**^**b**^	**9**^**b**^
Pre-treatment	**LP**	4.43*	8.43	5.71*					
	**ILP**	9.57+	7.86+	7.14+					
	**TC**	**14.00**	**16.29**	**12.86**					
Post-treatment	**LP**	9.00N	6.00		10.00	25.86+	15.86+	10.43N	16.86+
	**ILP**	3.86N	8.86+		1.71*			5.29N	
	**TC**	**12.86**	**14.86**		**11.71**	**25.86**	**15.86**	**15.71**	**16.86**
	**LP**	5.57*	8.71N		10.14			10.57	
	**ILP**	2.43*	6.00N		0.71*			2.14*	
	**TC**	**8.00**	**14.71**		**10.86**			**12.71**	
	**LP**	9.43N			12.14				
	**ILP**	3.71N			2.43*				
	**TC**	**13.14**			**14.57**				

^a^*LP* = Luteal phase; *ILP* = Inter-luteal phase; *TC* = Total cycle length.

^b^GnRH treatment.

^c^Control.

* = short phase; + = long phase; N = normal cycle.

For the Upper Escarpment herd, the low sample numbers collected from the four females, led to large gaps in the weekly data which limited any inferences regarding oestrous cycle phases.

### Effect of seasonality on FPM concentration

FPM concentrations in samples collected from all individual females during the wet season (median = 4.03, IQR = 3.42–4.67) were significantly higher than those of samples collected during the dry season (median = 2.59, IQR = 1.88-3.15; z = -78; n = 12, p<0.002).

**Figure 2:**
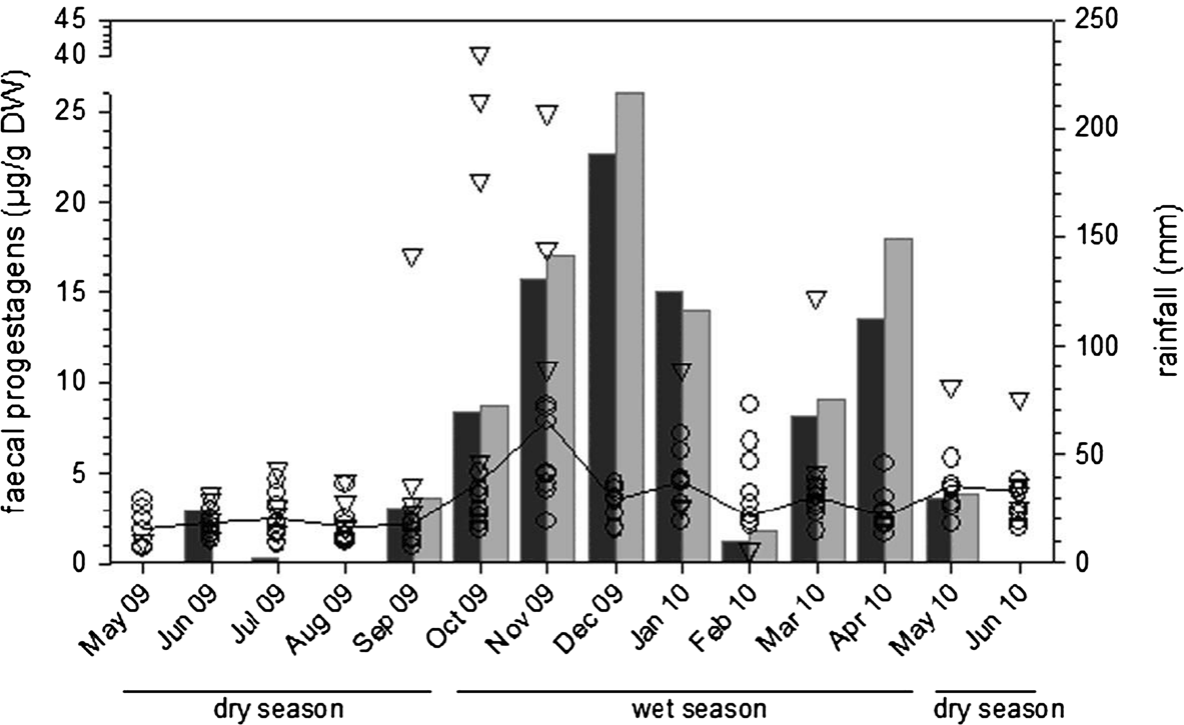
**Scatter plot of faecal progesterone metabolite concentrations from 12 female African elephants during one-year study.** Circles represent individuals from the Lower Escarpment herd (n = 8) and triangles represent individuals from the Upper Escarpment herd (n = 4). Line represents overall mean monthly FPM concentration. Vertical bars represent mean monthly rainfall corresponding to the study period for the Lower Escarpment (dark grey bars) and Upper Escarpment (light grey bars).

As there was no effect of treatment on FPM concentrations between groups, the treatment and control females were pooled to quantify the effect of season on FPM concentrations. In general, the average monthly FPM concentrations closely followed variations in rainfall patterns, with lower concentrations during the dry season and higher concentrations during the wet season. For the Upper Escarpment herd few samples were collected during the wet season, although the increase in average concentrations in these was noteworthy (Figure 2).

## Discussion

In this study two doses of 600 μg GnRH vaccine failed to induce anoestrus in female elephants as was anticipated. The apparently ineffective or incomplete action of the vaccine was possibly a consequence of under-dosing or the booster was administered too late to down-regulate the second cycle in some females that had already entered their luteal phase before treatment, as the stage of the oestrous cycle was unknown at the time of vaccination. An additional possibility might be incomplete delivery of the treatment due to the viscous nature of the solution. The long oestrous cycle of the African elephants [12,13] and the effect of the variability of the ecological conditions on FPM concentrations [10,11] made it difficult to elucidate the effect of the GnRH vaccine treatment on the oestrous cycle over the 12 month monitoring period in this study. We would recommend extending the observation period to two to three years in sites with seasonal variation and additionally using a progestagen EIA method to assess in-the-field ovarian activity as described by Freeman ([2011) [53] in order to optimize the vaccination by applying it during the luteal phase [23]. It is reported that five adult captive elephant cows (four of which had calved previously) treated in Zimbabwe with the same vaccine and dose as a means of contraception did not show behavioural oestrus for a period of two years following treatment (Bertschinger, personal communication). The cows that were housed with three adult bulls were initially vaccinated three times at intervals of 5 weeks. This was followed by boosters administered every 6 months. Differences that could account for the positive response include the additional initial booster and administration of vaccine by hand-injection.

All 12 monitored females showed some evidence of ovarian cyclicity during the 14-month study, although most (75%) of the cycles did not fall within the reported normal 13-17 weeks oestrous cycle range reported for African elephants [13]. Three (2 treatment, 1 control) females from the Lower Escarpment showed a period of anoestrus which after the vaccination and onset of the rainy season was followed by a prolonged luteal phase (25.86, 15.86 and 16.86 weeks, respectively) (Table 2). The Upper Escarpment herd had higher mean luteal, inter-luteal and peak FPM concentrations compared to the Lower Escarpment herd in both treatment and control group, which may be attributed to the different habitats and the resource availability, more pronounced in the Upper Escarpment through the seasons with a higher average rainfall and nutritionally poorer vegetation compared to the Lower Escarpment. Factors including the lack of changes in group dynamics, absence of mature males and a fenced environment preventing seasonal movements may have contributed to the high incidence of cyclic abnormalities observed in EPGR elephant herds. The potential effects of pZP vaccine on variability of the oestrous cycles of the four treated cows (numbers 1, 2, 8, 9) cannot be completely excluded. All four cows showed luteal activity (Cow 1: Figure 1 a) and their LP and ILP FPM concentrations were similar to those of their herd mates on the Lower Escarpment. As reported by Ahlers *et al.* ([2012) [19], ovarian function did not appear to be disrupted by pZP treatment.

In wild elephants, we presume that sexually-receptive periods in females result in mating and pregnancy. These females subsequently will not cycle again two to three years after a gestation period of 22 months [34,35,54]. Repeated oestrous cycles are thus an abnormal feature of reproduction in free-ranging elephants [7,55,56]. Abnormal cycles in female elephants are currently not well understood [57]. With the exception of reports by Ahlers *et al.* ([2012) [19] and Ghosal *et al.* ([2012) [58], limited information is available which our data can be compared to. In a one-year study conducted on a wild herd of African elephants in South Africa 42.9% of females treated with pZP vaccine failed to cycle and 14.3% had irregular cycles [19]. Three distinctive types of hormone profiles were observed in semi-captive Asian elephant (*Elephas maximus*) females [58]. These were normal oestrous cycles, acyclic patterns or progestagen profiles with high values indicative of a pregnant animal. In captive populations in North America, 43% of African elephants also showed abnormal cycles. The proposed causes in these captive elephants include reproductive tract pathology, alteration in the secretion of pituitary gonadotropins and thyroid hormones and hyperprolactinaemia [57,59]. Other studies report social and environmental variables affecting ovarian cyclicity[57,60].

The effect of seasonal changes on reproductive hormonal activity in non-pregnant free-ranging African elephants has been previously documented [10,11,19]. In our study the effect of the dry season (starting May through September) on FPM levels was evident in nearly all study animals (6 treatment, 4 control). They either had lower FPM levels or a period of hormonal flat-lining (anoestrus). In the latter case cyclic activity resumed during the wet season (October through April). A comparison of FPM concentrations for the three-month period before and after treatment revealed a significant increase. This effect, however, was present in treated and control individuals and most probably can be attributed to the onset of the rainy season. The four Upper Escarpment females (Females No. 11, 12, 13, and 14) as well as four females (Females No. 4, 5, 7, and 8) from the Lower Escarpment appeared to experience a period of seasonal anoestrus during the dry season 2009. Following the onset of the subsequent dry season (post-treatment) in May 2010, the FPM levels decreased, and in almost all treated animals (5 of 6; Females No. 1, 2, 3, 8, and 9) from the Lower Escarpment herd, the levels were lower than the previous dry season. This might reflect either an incomplete effect of the GnRH vaccine causing a reduced secretion of progestins, or lower rainfall received during the previous season resulting in limited abundance of resources. In general, the average monthly FPM concentrations closely followed rainfall patterns and confirmed the relationship reported in the literature between ecological variation and ovarian activity [10,11,19]. Thus African elephants appear to optimize the timing of oestrus in order to maximize use of seasonal availability of resources to coincide with the energetic investment needed for reproduction [61].

## Conclusions

This study concluded that a primary dose of 600 μg GnRF-protein conjugate followed by a single booster of the same dose, five to seven weeks later failed to induce anoestrus in eight adult female African elephants. A review of the vaccination protocol, as well as a longer observation period is recommended for future studies. The ecological factors affecting reproduction can be applied to enhance the efficacy of the contraceptive by optimizing the timing of administration to achieve the highest population response, which in this case (EPGR; summer rainfall and dry winter), should be most effective if applied at the onset of the dry season.

The results of this study showed the ovarian endocrine activity of non-pregnant female African elephants and confirm that abnormal oestrous cycles are a common feature amongst wild elephants in the absence of adult bulls [19] and is not restricted or attributable to captivity as anticipated by several authors [13,43,57,59,60,62,63]. Our findings also indicate that female elephants do not cycle continuously in the absence of adult bulls. These observations provide important information on endocrine function and reproductive activity that can be applied to improving elephant population management and control.

## Abbreviations

DW: Dry weight; EIA: Enzyme immunoassay; EPGR: Entabeni Private Game Reserve; FPM: Faecal progestagen metabolites; FSH: Follicle stimulating hormone; GnRH: Gonadotropin releasing hormone; ILP: Inter-luteal phase; LH: Luteinising hormone; LP: Luteal phase; pZP: Porcine zona pellucida; GnRF: Gonadotropin releasing factor; VHF: Very high frequency.

## Competing interests

The authors declare that they have no competing interests.

## Authors’ contribution

GBV was responsible for data collection, analysis, interpretation, and drafting of the manuscript. AG coordinated the laboratory analysis of faecal samples and helped to draft the manuscript. AG, MLS and HA assisted in the analysis and interpretation of data and helped to draft the manuscript. HJB conceived, participated in the design and coordination of the study, was involved in the implementation of the field work and helped to draft the manuscript. All authors read and approved the final manuscript.

## Supplementary Material

Additional file 1 **Figure S1.**Faecal Progestagen Concentrations. Faecal progestagen metabolite concentrations for 12 adult female African elephants. Red solid line represents baseline concentration, horizontal solid bars represent luteal phase, horizontal open bars represent inter-luteal phase, and dotted line arrows correspond to darting dates of primary vaccine and subsequent booster vaccine on treated individuals (and only as a reference on control individuals). Circles stand for sniffing into genital opening or urine and stars for vaginal discharge observations. Wet season is illustrated in blue background. Click here for file
