# Supplementary material for: Non-invasive assessment of the reproductive cycle in free-ranging female African elephants (Loxodonta africana) treated with a gonadotropin-releasing hormone (GnRH) vaccine for inducing anoestrus
Source: Reprod Biol Endocrinol. 2012 Aug 25;10:63. doi: 10.1186/1477-7827-10-63 (PMC3485135; doi:10.1186/1477-7827-10-63)
Supplement: Additional file 1 — Figure S1.Faecal Progestagen Concentrations. Faecal progestagen metabolite concentrations for 12 adult female African elephants. Red solid line represents baseline concentration, horizontal solid bars represent luteal phase, horizontal open bars represent inter-luteal phase, and dotted line arrows correspond to darting dates of primary vaccine and subsequent booster vaccine on treated individuals (and only as a reference on control individuals). Circles stand for sniffing into genital opening or urine and stars for vaginal discharge observations. Wet season is illustrated in blue background. [file 1477-7827-10-63-S1.pdf]

Additional figures: Faecal progestagen metabolite concentrations for 12 adult female African elephants. Red solid line represents baseline concentration, horizontal solid bars represent luteal phase, horizontal open bars represent inter-luteal phase, and dotted line arrows correspond to darting dates of primary vaccine and subsequent booster vaccine on treated individuals (and only as a reference on control individuals). Circles stand for sniffing into genital opening or urine and stars for vaginal discharge observations. Wet season is illustrated in blue background.

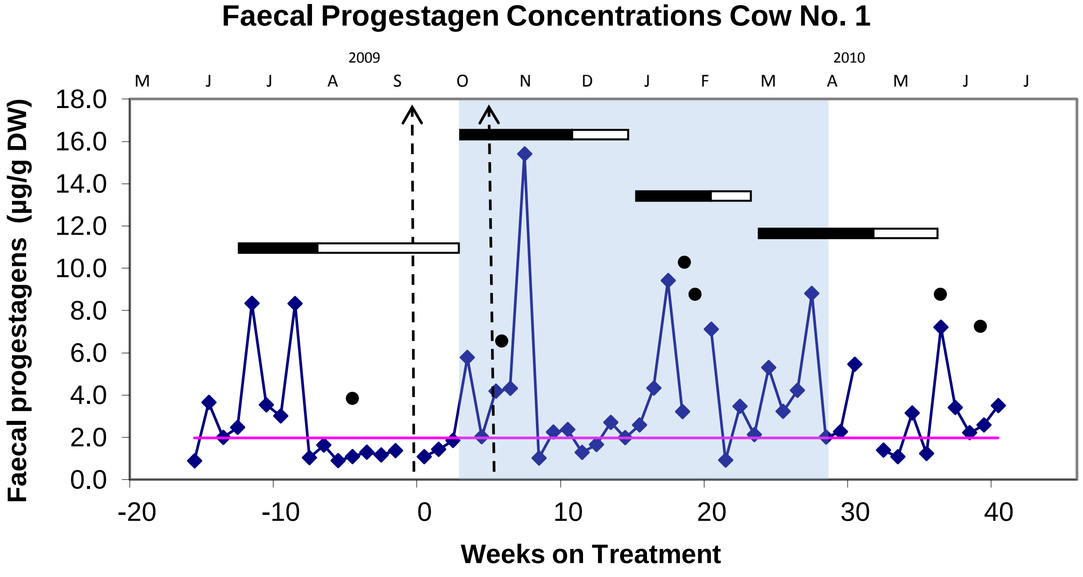

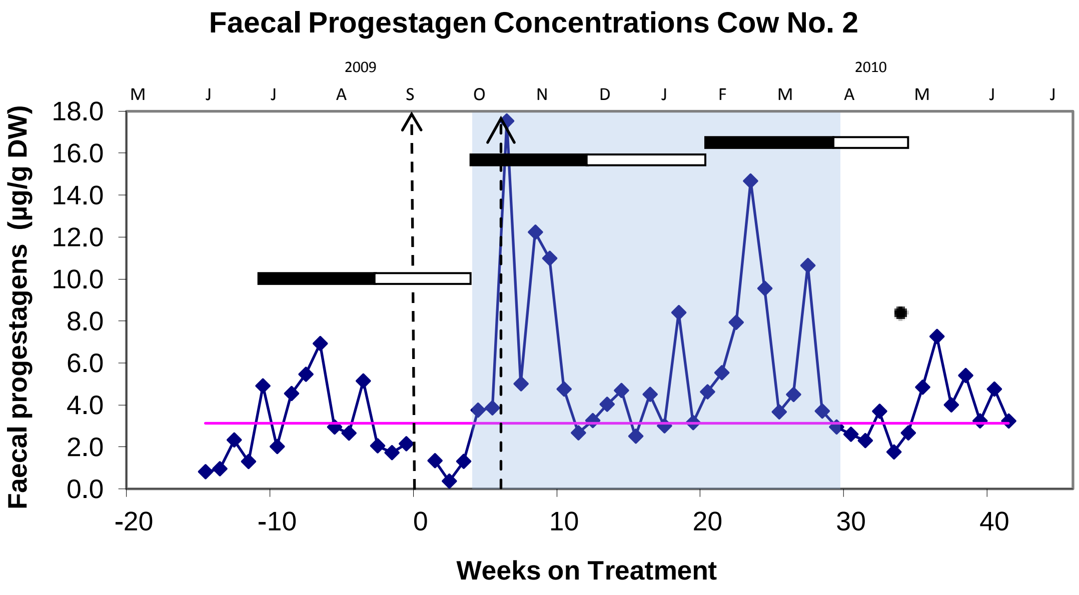

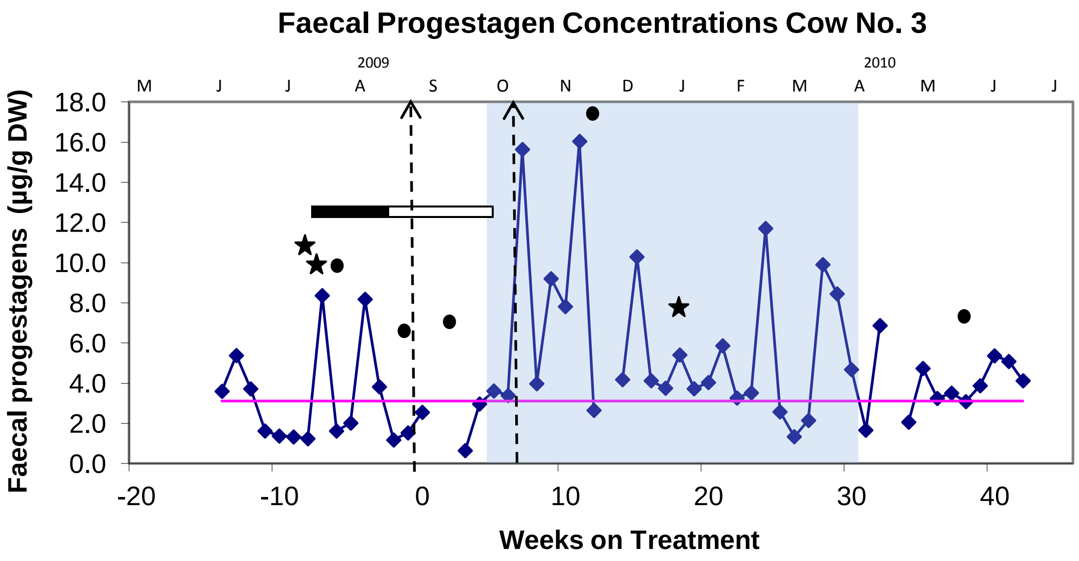

Faecal Progestagen Concentrations Cow No. 3
2009
2010
M J J A S O N D J F M A M J J
Faecal progestagens (µg/g DW)
18.0
16.0
14.0
12.0
10.0
8.0
6.0
4.0
2.0
0.0
-20
-10
0
10
20
30
40
Weeks on Treatment

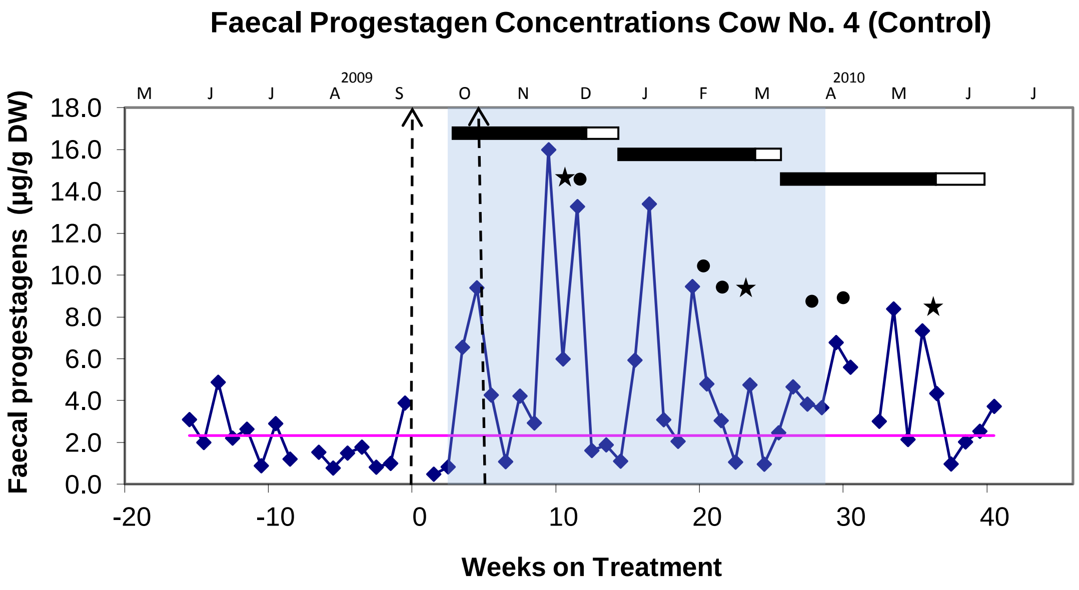

Faecal Progestagen Concentrations Cow No. 4 (Control)
2009
2010
M J J A S O N D J F M A M J J
Faecal progestagens (µg/g DW)
18.0
16.0
14.0
12.0
10.0
8.0
6.0
4.0
2.0
0.0
-20
-10
0
10
20
30
40
Weeks on Treatment

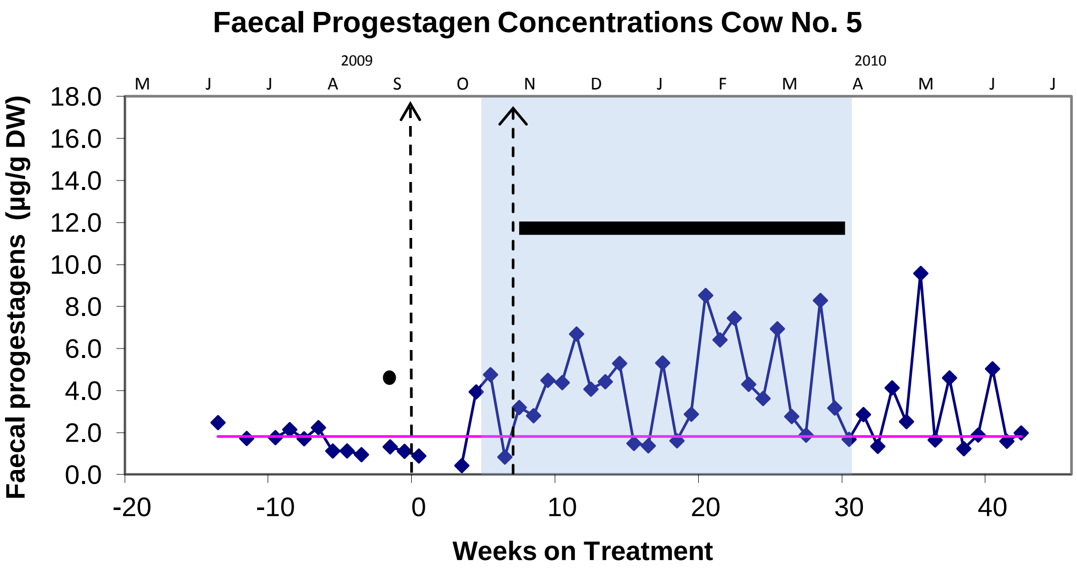
Faecal Progestagen Concentrations Cow No. 5
2009
2010
M J J A S O N D J F M A M J J
Faecal progestagens (μg/g DW)
18.0
16.0
14.0
12.0
10.0
8.0
6.0
4.0
2.0
0.0
-20
-10
0
10
20
30
40
Weeks on Treatment

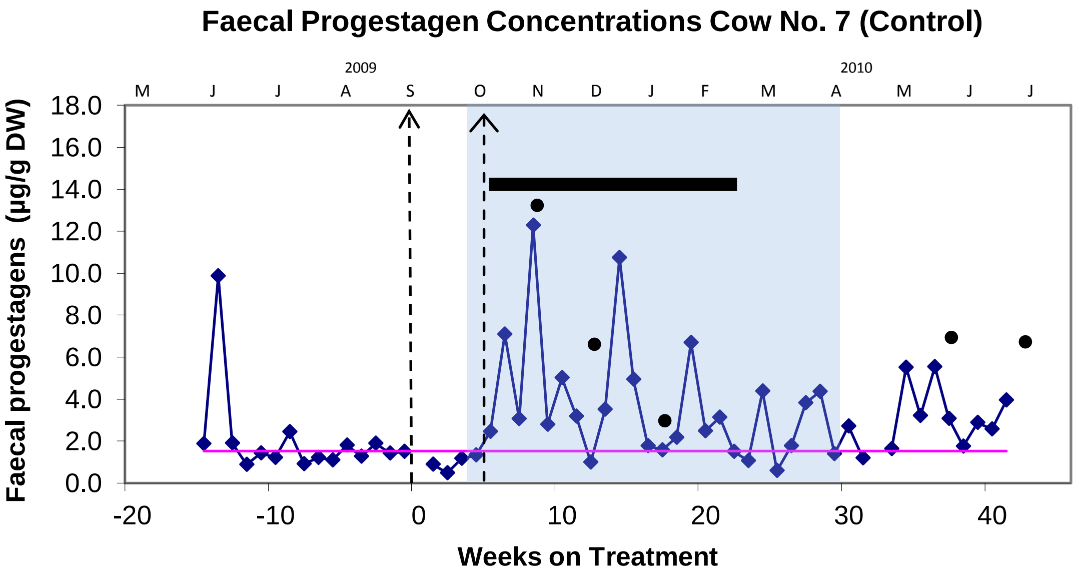
Faecal Progestagen Concentrations Cow No. 7 (Control)
2009
2010
M J J A S O N D J F M A M J J
Faecal progestagens (μg/g DW)
18.0
16.0
14.0
12.0
10.0
8.0
6.0
4.0
2.0
0.0
-20
-10
0
10
20
30
40
Weeks on Treatment

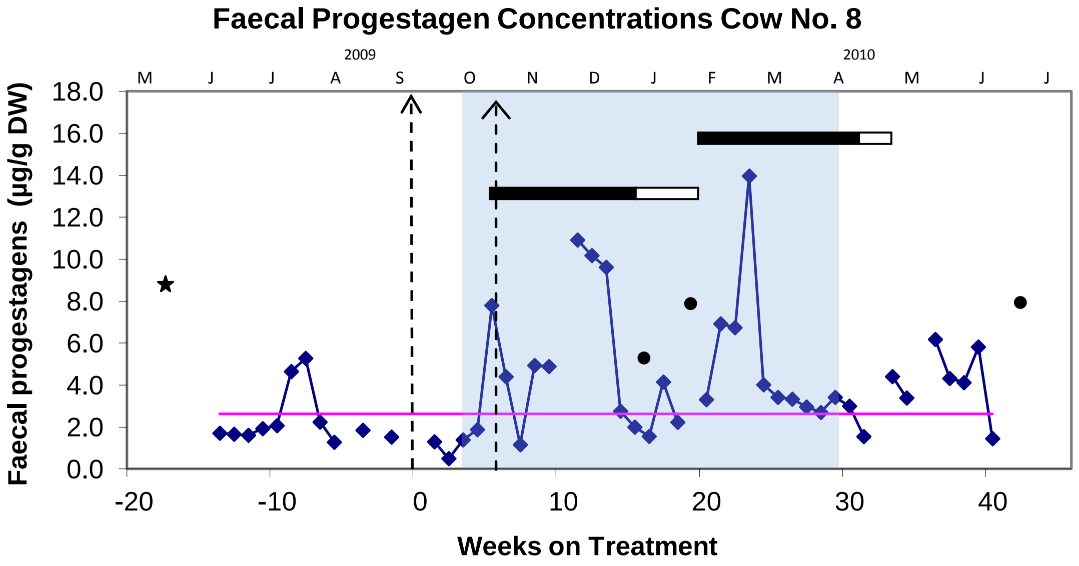
Faecal Progestagen Concentrations Cow No. 8
2009
2010
M J J A S O N D J F M A M J J
Faecal progestagens (µg/g DW)
18.0
16.0
14.0
12.0
10.0
8.0
6.0
4.0
2.0
0.0
-20 -10 0 10 20 30 40
Weeks on Treatment

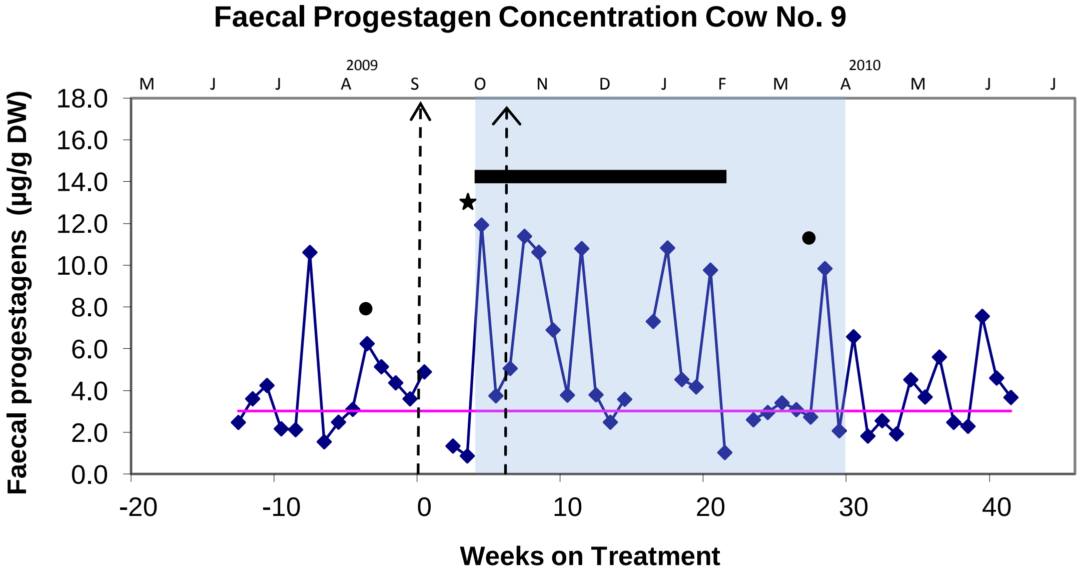
Faecal Progestagen Concentration Cow No. 9
2009
2010
M J J A S O N D J F M A M J J
Faecal progestagens (µg/g DW)
18.0
16.0
14.0
12.0
10.0
8.0
6.0
4.0
2.0
0.0
-20 -10 0 10 20 30 40
Weeks on Treatment

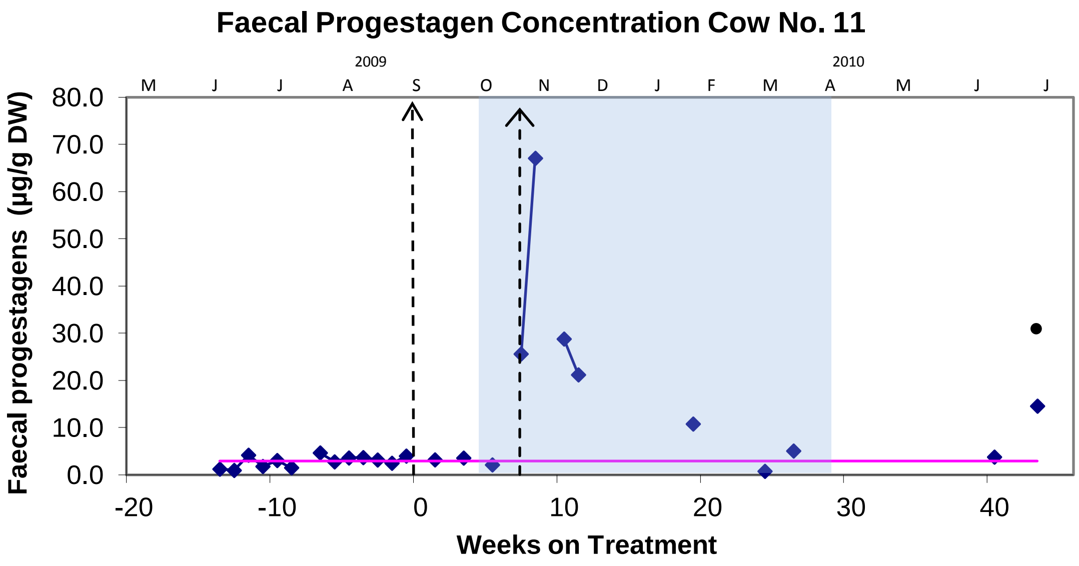

Faecal Progestagen Concentration Cow No. 11
2009
2010
M J J A S O N D J F M A M J J
Faecal progestagens (µg/g DW)
80.0
70.0
60.0
50.0
40.0
30.0
20.0
10.0
0.0
-20
-10
0
10
20
30
40
Weeks on Treatment

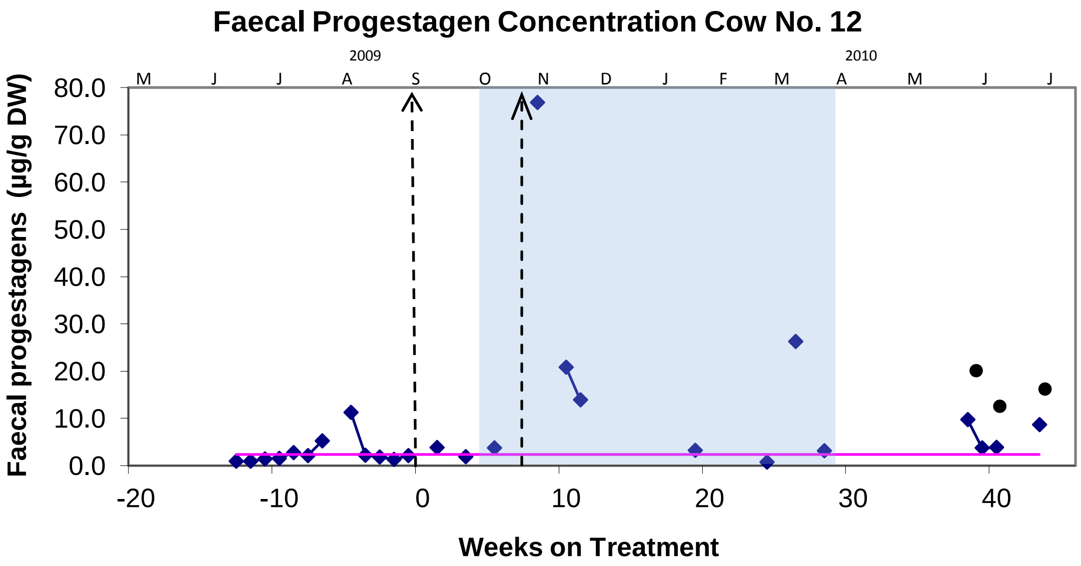

Faecal Progestagen Concentration Cow No. 12
2009
2010
M J J A S O N D J F M A M J J
Faecal progestagens (µg/g DW)
80.0
70.0
60.0
50.0
40.0
30.0
20.0
10.0
0.0
-20
-10
0
10
20
30
40
Weeks on Treatment

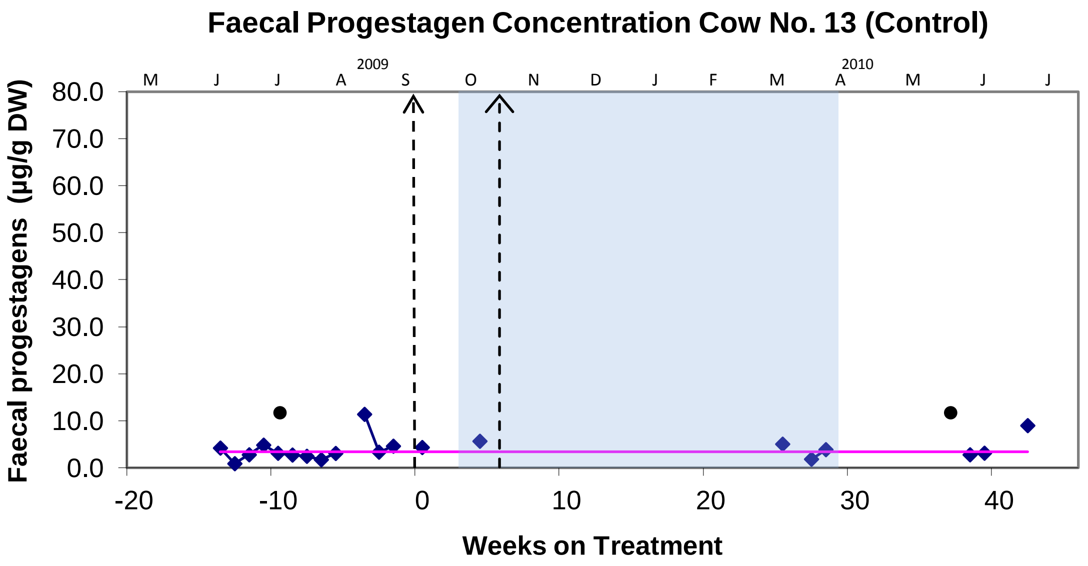

Faecal Progestagen Concentration Cow No. 13 (Control)
2009
2010
M
J
J
A
S
O
N
D
J
F
M
A
M
J
J
Faecal progestagens (µg/g DW)
80.0
70.0
60.0
50.0
40.0
30.0
20.0
10.0
0.0
-20
-10
0
10
20
30
40
Weeks on Treatment

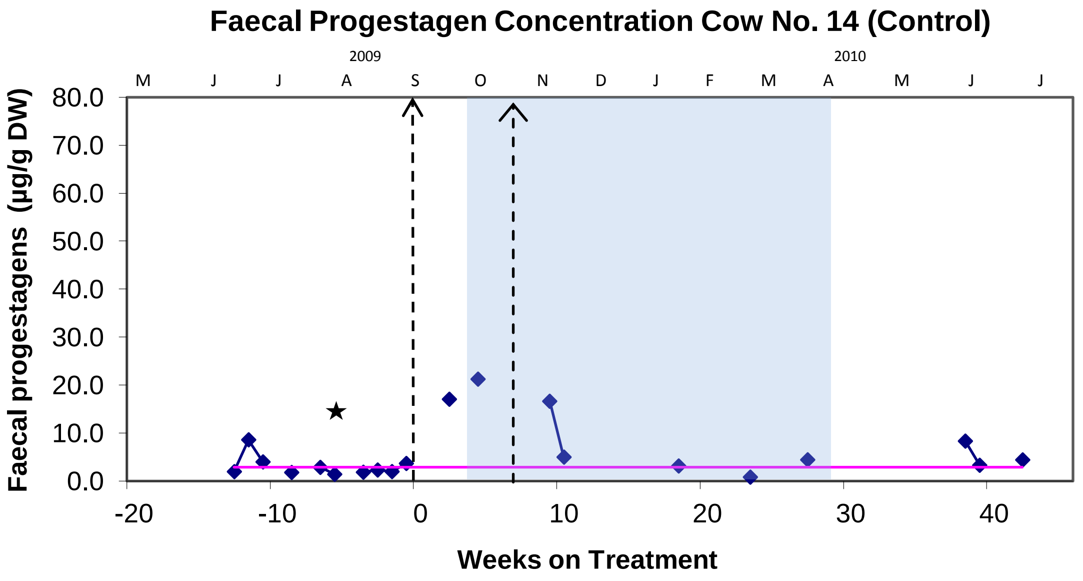

Faecal Progestagen Concentration Cow No. 14 (Control)
2009
2010
M
J
J
A
S
O
N
D
J
F
M
A
M
J
J
Faecal progestagens (µg/g DW)
80.0
70.0
60.0
50.0
40.0
30.0
20.0
10.0
0.0
-20
-10
0
10
20
30
40
Weeks on Treatment
